# Optimization of the Dry Turning Process of Ti48Al2Cr2Nb Aluminide Based on the Cutting Tool Configuration

**DOI:** 10.3390/ma15041472

**Published:** 2022-02-16

**Authors:** Enrique García-Martínez, Valentín Miguel, Alberto Martínez-Martínez, Juana Coello, Jesús Andrés Naranjo, María Carmen Manjabacas

**Affiliations:** 1 High Technical School of Industrial Engineers of Albacete, University of Castilla-La Mancha, 02071 Albacete, Spain; enrique.gmartinez@uclm.es (E.G.-M.); juana.coello@uclm.es (J.C.); jesus.naranjo@uclm.es (J.A.N.); mcarmen.manjabacas@uclm.es (M.C.M.); 2 Regional Development Institute, Science and Engineering of Materials, University of Castilla-La Mancha, 02071 Albacete, Spain; alberto.martinez@uclm.es

**Keywords:** titanium aluminide, sustainable machining, coated and uncoated tools, tool wear, surface integrity

## Abstract

Titanium aluminides are one of the most promising materials in aeronautical and automotive applications. However, their low machinability makes the processing of these alloys quite difficult under sustainability conditions, specially without lubrication. The current study focuses on the turning process of the Ti48Al2Cr2Nb gamma titanium aluminide under dry conditions. As far as we are aware, dry cutting is the most sustainable feature, although it has not been traditionally applied on titanium aluminides due to the accelerated tool wear that the material promotes. The main novelty of this work consists of providing a simple solution for reducing the tool wear based on the inclination of the cutting insert, what is evaluated in terms of tool wear and tool life, cutting forces, cutting temperature, surface integrity of the machined part, as well as its microhardness and microstructural effects. The results shown here clearly point out a better performance of the machining process. This fact could be understood if we take into consideration that an increment of the clearance angle from 6.3° to 11.6° and 15° increases the tool life by five and six times, respectively, using efficient cutting speeds, whose values have increased by 50% with respect to the original cutting conditions. This improvement is explained according to the reduction in the cutting temperature and friction forces in the flank face of the tool. In addition, the use of uncoated carbide inserts may lead to a better behaviour than the coated ones, considering the results obtained for a PVD TiAlN + AlCr_2_O_3_ coated insert herein researched.

## 1. Introduction

Among the materials developed in recent decades, titanium aluminides have been identified as potential candidates for aeronautical and automotive applications [1]. These alloys are considered substitutes for Inconel-718 or Ti6Al4V for parts subjected to high temperatures. This is due to their interesting properties consisting of having lower density and a higher strength-to-weight ratio compared with Inconel-718. In addition, their higher strength at great temperatures and better corrosion and oxidation resistance in comparison with Ti6Al4V should not be underestimated [2]. The most promising alloys are found in the range of 40–48 aluminium atomic percentage, being formed by α_2_(Ti_3_Al) and γ(TiAl) phases [3]. Different microstructures such as near-γ, duplex, nearly lamellar or fully lamellar can be obtained depending on the heating treatment applied to the material, leading to variations in the mechanical and processing properties of these aluminides [4]. However, the applicability of Ti-aluminides is limited due to their low ductility and fracture toughness at temperatures below 600–800 °C, which correspond to the brittle–ductile transition of those materials. On the other hand, production costs for these alloys are higher than for nickel-based superalloys due to the lack of processing techniques for materials with low ductility [5]. In addition, the manufacture of titanium aluminide parts becomes difficult to carry out by processes such as extrusion, forging or similar ones. Thus, casting, ingot metallurgy, powder metallurgy and, recently, additive manufacturing have become much more suitable methods. However, the difference among the melting points of the components, and the difficulty in controlling the cooling rates, leads to heavy segregation and non-uniform structures in the parts manufactured by casting and ingot metallurgy [6]. Usually, some thermo–mechanical treatments or hot-isostatic pressing (HIP) steps are required to reduce porosity and improve chemical homogeneity [1]. Recently, electron beam melting (EBM) has been identified as a potential additive manufacturing technology for the manufacture of complex titanium aluminide parts, usually followed by the HIP process [7]. Nevertheless, the surface quality obtained by all these manufacturing methods is frequently poor. For example, values of 54 µm for Ra are typically obtained for parts made by electron beam melting (EBM) [8]. Therefore, some finishing machining processes are mandatory to improve the surface quality of the parts.

However, titanium aluminides have been reported as difficult-to-machine materials because of their high hardness, low thermal conductivity and high chemical affinity with tool materials, which results in excessive cutting temperatures [9]. Castellanos et al. [9] established that thermal gradients, combined with high cutting forces and mechanical loads, produce accelerated tool wear along with a strong work-hardening tendency, which affects the surface integrity and results in subsurface alterations. Aspinwall et al. [10] reported that the turned workpieces show surface smearing, cracks and subsurface lamellae deformation, which is a proven sign of the existence of strain-hardening at high temperatures. They found that very low cutting speeds (about 20–25 m/min) were required to perform the process under a stable behaviour condition, and to control tool wear.

Some studies have been carried out on titanium aluminide turning, searching for the most efficient cutting conditions. Recently, Peng and Shareef [11] have analysed the turning process of a γ-TiAl alloy for a wide range of cutting conditions, stating the difficulty of the machining operations, which can only be conducted at low speeds if typical feed rates are applied. In relation to this, Chowdhury et al. [12] reported that a cutting speed of 60 m/min is adequate if a low feed rate (0.05 mm/rev) is used, although a coolant flow rate of 8 L/min is required to perform the machining operation. On the other hand, Klocke et al. [13] found that machining at a cutting speed of 100 m/min, which is within the range of high-speed turning, improved chip formation, since the high temperatures reached in the cutting zone contributed to the production of a thermal softening effect. However, although this improvement is promising, they reported that an effective cooling strategy is needed to develop the operation at that cutting speed, due to the observation of the rapid degradation of the tool. These conditions lead to an extremely low tool life, which makes the process nonoperative.

Other efforts aim for the possibility of improving the machinability of the Ti-aluminides by using an efficient lubrication. Moreover, considering that traditional lubrication involves environmental and health risks, causing, for instance, respiratory diseases, it is necessary to look for efficient lubrication methods in the field of the machining of titanium aluminides. Klocke et al. [14], established that cryogenic lubrication was the most promising cooling strategy for Ti-aluminides machining because it could counteract the thermal loads generated during the cutting process. On their part, Priarone et al. [15] studied the turning process of Ti48Al2Cr2Nb aluminide under minimum quantity lubrication (MQL) and low cutting fluid (water and emulsion), finding that both methods admitted only reduced cutting parameters, since their cooling capacity was insufficient. Moreover, flood lubrication did not lead to significant improvement.

Regarding the tool material, Priarone et al. [16] evaluated the tool life and the surface integrity on the turning process of Ti-aluminides with polycrystalline diamond (PCD), cubic boron nitride (CBN), and uncoated and coated carbide tools, under wet-cutting and cryogenic conditions. They found an optimal value of 30 min for a PCD tool life under cryogenic conditions, for a cutting speed of 80 m/min and a feed rate of 0.1 mm. However, the applicability of PCD tools is limited due to their expensive cost in comparison with carbide tools, and their underperformance in the case of vibration phenomena.

The efficiency of coated and uncoated cutting inserts for the turning of Ti-aluminides is still being studied, as it is necessary to find effective methods to reduce the surface defects of the parts, increasing the tool life at the same time. From a tribological viewpoint, García-Martínez et al. [17] recently evaluated the performance of coated and uncoated carbide tools under sliding friction against Ti48Al2Cr2Nb aluminide at different temperatures. They reported that an uncoated tool and a PVD TiAlN + AlCr_2_O_3_ coated one could be the most suitable inserts for turning the γ-TiAl alloy. They also found that cryogenic lubrication with gaseous nitrogen at −50 °C was not effective in terms of friction coefficient. Yao et al. [18] analysed the surface integrity and the fatigue behaviour of turned parts by different coated carbide tools. They used PVD-TiCN and PCD-TiAlN coated tools, finding that a PVD-TiCN coated tool exhibited the best behaviour. Recently, Anwar et al. [19] studied the machinability of an electron-beam-melted γ-TiAl by using standard coated and uncoated carbide inserts for a wide range of cutting speeds, within 40 m/min and 80 m/min. They analysed the involved cutting forces, the surface roughness obtained, and the tool wear mechanisms involved and found that the uncoated tool behaved better than the coated one. However, the tool life was not considered in that research.

Among the environmentally friendly methods, dry cutting is the most sustainable technique thanks to the non-use of any coolant or oil [20]. Thus, efficient strategies to improve tool life, making machining more efficient, are necessary to investigate when a dry condition is selected for the turning processes of titanium aluminides. However, there is a lack of studies on the dry machining of titanium aluminides that look for solutions to increase tool life and make the process more suitable for the industry.

In the present research, a simple solution is shown for the turning process of Ti-aluminide Ti48Al2Cr2Nb, based on the tool inclination, which represents the main novelty of the work. The effect of increasing the clearance angle between the flank face of the tool and the workpiece is evaluated in terms of tool wear morphology and tool life, cutting forces, cutting temperature, surface roughness, subsurface hardness, and microstructure alterations of the workpiece. The analysis was developed for a wide range of cutting speeds within 40–70 m/min, by using an uncoated carbide insert and a coated one with PVD-TiAlN-AlCr_2_O_3_.

## 2. Materials and Methods

### 2.1. Workpiece Material

Machinability investigations were performed on gamma Ti-aluminide Ti48Al2Cr2Nb, whose chemical composition is indicated in Table 1. The parts were melted via a vacuum-arc furnace from consumable electrodes and subsequently hot-isostatic-pressed, HIP, obtaining cylinders of 57 mm diameter and 25 cm long.

**Figure 1:**
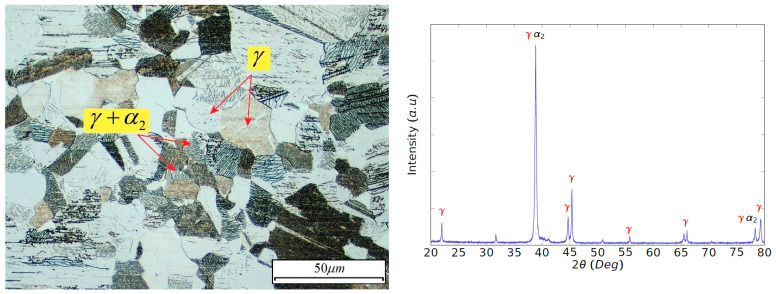
(**a**) Microstructure and (**b**) XRD pattern of the Ti48Al2Cr2Nb alloy.

Samples for microstructural analysis were cut by wire electrical-discharge machining, wire-EDM, in order to avoid high thermal gradients during the cutting operation. The samples were first ground, later polished, and finally etched for 5–10 s with a solution of 1 mL of HF, 1 mL of HNO_3_ and 1 mL of acetic acid in 100 mL of solution volume. The microstructure of the Ti-aluminide alloy is shown in Figure 1a and consists of equiaxed γ-phase grains (TiAl) and lamellar colonies of the phases γ and α_2_ (Ti_3_Al). Analysis through X-ray diffraction, XRD, of the sample revealed the existence of more intense peaks of γ-phase than α_2_-phase, as can be seen in Figure 1b.

### 2.2. Experimental Methodology

**Figure 2:**
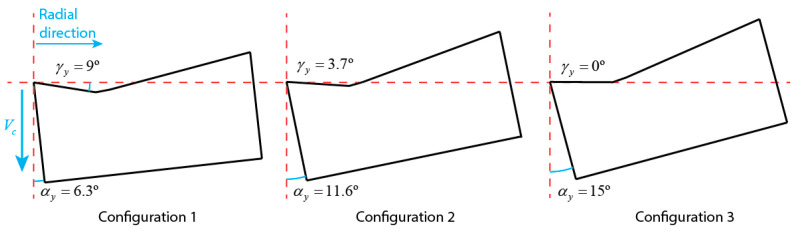
Schematic detail of the tool angles for each configuration.

The Ti-aluminide cylinders were externally turned on a 10kW semi-automatic lathe Microcut H-2160. The lathe was equipped with a variable frequency drive to keep the cutting speed constant in each trial. A TNMG 160408-SM H13A uncoated carbide tool from Sandvik Coromant was selected based on the authors’ previous research [17]. An MTJNR 2525M 16M1 toolholder, from Sandvik Coromant, was used. Two additional steel shims were milled to evaluate the influence of the tool position on the cutting operation, which allowed the cutting angles to vary, as shown in Table 2. A guide system was used to obtain inclined planes. Later, the resulting angles in both axial and radial directions were measured by a FormTalysurf 50 profilometer (Taylor Hobson Inc., Leicester, UK). The resulting angles are the sum of the shim angles and the insert ones in both directions. This proposal definitely addressed the change of the inclination of the cutting insert, incrementing the clearance angle between the tool and the workpiece, as well as reducing the rake angle, as can be observed in Figure 2.

The workpieces were subjected to preliminary roughing operations, with a depth of cut of 0.25 mm, a feed rate of 0.01 mm/rev and a cutting speed of 15 m/min, to remove the cast skin and to ensure a uniform diameter of the cylinders. For the machining tests, the depth of cut and the feed rate were fixed at 0.25 mm and 0.01 mm/rev, respectively, while the selected levels for the cutting speed were 40 m/min, 50 m/min, 60 m/min and 70 m/min, Table 3. Additional tests with the TNMG 160408-SM 1115 insert and PVD coated with TiAlN + AlCr_2_O_3,_ from Sandvik Coromant (SANDVIK Corp., Stockholm, Sweden) were developed, only at the cutting speed of 50 m/min, to provide a comparison with the uncoated tool. Finally, all tests were developed under dry conditions.

The machinability of the Ti-aluminide was evaluated in terms of tool wear, tool life, cutting forces, cutting temperature, surface roughness, surface integrity and subsurface alterations. According to the cylinder dimensions, the maximum usable working length in the axial direction was 135 mm. Machining passes of approximately 200 m cutting length were carried out under each cutting condition combination until the end of the tool life, obtaining a continuous description of the flank wear. In the middle of each trial, the feed rate was interrupted for 10 s with the objective of evaluating the forces and temperature due only to the friction phenomenon in the tool–workpiece contact. Consequently, during this time there was no chip removal. Consistent and uniform values were always recorded, following a logical evolution of the test, with no anomalous, out-of-trend or noisy results, which established a high reliability of the measurement procedure.

The three main components of the cutting force and their evolution were registered by using a three-axis dynamometer, built by the research team, with a capability of measuring forces up to 5000 N and a resolution lower than 1 N. The dynamometer is composed for four strain-gauge load cells that were calibrated under static conditions and validated in previous studies [21]. The temperature for cutting operations with uncoated WC inserts was registered by using a natural workpiece-thermocouple technique formed by the contact pair workpiece–tool, that is, Ti48Al2Cr2Nb-WC/Co, verified for this kind of application in previous research [22]. According to the calibration data, the uncertainty of the instrument could be fixed in the range 7–8% within the range of temperatures involved in this research.

The surface roughness at the early beginning of the turning operation with a new tool was analysed by using a three-dimensional roughness profilometer FormTalysurf50 from TaylorHobson and processed using the specific Talymap Gold software (6.0, Taylor Hobson Inc., Leicester, UK). A random rectangular area of 5 × 4 mm^2^ was selected for the measurements. An Olympus SZX7 stereomicroscope was used to evaluate tool wear after each cutting trial by controlling the increment of the flank wear band, *V_b_*. The end of the tool life was established for a flank wear value *V_b_* of 0.100 mm, for which a significant worsening of the surface integrity of the machined cylinders was observed. In addition, the tool wear morphology was studied by a scanning electron microscope (SEM) Jeol 6490 LV (Jeol Ltd., Tokyo, Japan) and the chemical composition of the wear area was analysed by electron dispersive spectroscopy (EDS). To evaluate the surface integrity and the subsurface alterations induced by the machining operation, some samples were extracted from the machined cylinders by wire-EDM, avoiding any overheating of the samples. After that, the samples were prepared for microscopical analysis. Knoop microhardness tests with a load of 50 g were performed with Shimadzu type M apparatus (Shimadzu Corp., Kyoto, Japan) to evaluate the subsurface hardening. For that purpose, microhardness measurements were taken every 10 µm in the radial direction of up to 300 µm from the machined surface.

## 3. Results and Discussion

### 3.1. Tool Wear and Tool Life

**Figure 3:**
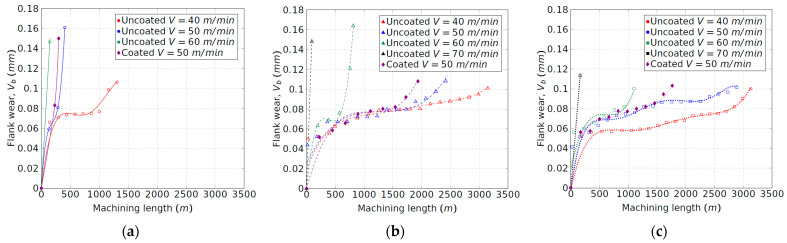
Tool wear evolution of the uncoated tool: (**a**) configuration 1; (**b**) configuration 2; and (**c**) configuration 3.

**Figure 4:**
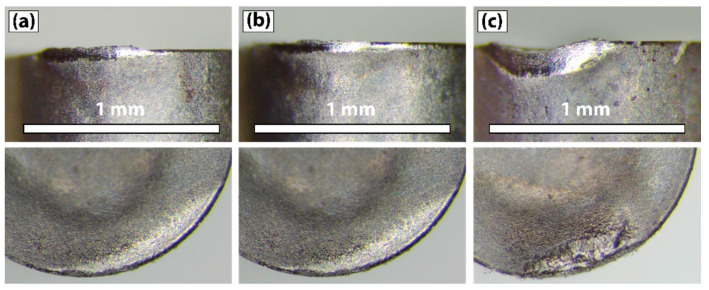
Flank and rake face wear of the uncoated tool after 120 m of machining length for configuration 1: (**a**) corresponds to 40 m/min; (**b**) to 50 m/min; and (**c**) to 60 m/min.

Figure 3 shows the tool wear evolution of the uncoated insert for each tool configuration and for the different cutting speeds experimented. The obtained results for the coated tool at 50 m/min are also shown. As expected, the cutting speed has a significant influence on tool wear. The increment of the cutting speed always promotes tool wear due to the increment of the cutting temperature, mostly in the flank wear zone, as will be discussed in the following sections. It was observed that, for the machining with configuration 1 (original shim), excessive heat was generated at 50 m/min and, especially, at 60 m/min. Reddish flashes and sparks coming out of the cutting zone were noticeable, increasing the speed of the abrasion phenomenon on the flank and on the rake faces of the tool, leading to severe crater wear (Figure 4). Figure 3a shows that tool wear develops rapidly at speeds of 50 and 60 m/min in comparison to 40 m/min. In the latter case, the flank wear gradually evolved, and the flashes and sparks, proof of the existence of high temperatures, were only visible above 1000 m of machining; this finally lead to the crater wear, which is consistent with the results of Priarone et al. [16]. In addition, at 70 m/min, the tool dramatically broke at the first contact with the workpiece. Thus, this case has not been represented in Figure 3a.

The effect of the tool inclination increment, that is, tool configurations 2 and 3, is observed in Figure 3b,c. A remarkable reduction in tool wear for all cutting speeds is found as the clearance angle is increased. The increment of that angle reduces the friction forces between the tool and the workpiece at the flank zone and prevents the temperature from rising in this area. Moreover, with tool configurations 2 and 3, no flashes or sparks were observed. Hence, the combination of the high mechanical and thermal loads is avoided, and with it, the cause of the tool damage in the original configuration. Thus, the machining length, which can be turned at 50 m/min, exhibits a remarkable increment. Therefore, for the uncoated tool and configuration 1, the machining length evolves from less than 500 m to nearly 2500 m and 2900 m for configurations 2 and 3, respectively. It means that, with the inclination of the tool, dry turning at a cutting speed of 50 m/min becomes possible in terms of cutting tool efficiency. The wear curves show a relative-constant zone in which the wear rate is under control. Comparatively, Priarone et al. [15] found a maximum machining length of 2000 m by using an emulsion mist lubricant for a cutting speed of 50 m/min. The results presented herein are promising and indicate that a better tool life could be obtained by using an adequate lubrication method if it is combined with appropriate configuration of the tool. The maximum machining length at a cutting speed of 40 m/min is the same for both configurations, 2 and 3. It means that the increment of the clearance angle shows no further effect, although the flank temperature is reduced, as has been discussed before. This result is consistent with the theory that abrasive and adhesive wear processes are predominantly determined by cutting length, while the processes of diffusion and oxidation, thermally activated, are governed by temperature [23].

Comparing both tools, the coated tool behaves worse than the uncoated tool for each configuration (see Figure 3). The coated layer, whose thermal conductivity is lower than the uncoated one [24], probably promotes high thermal gradients. Furthermore, no improvement from configuration 2 to configuration 3 is found for this tool, the maximum machining length being less than 2000 m under those conditions.

**Figure 5:**
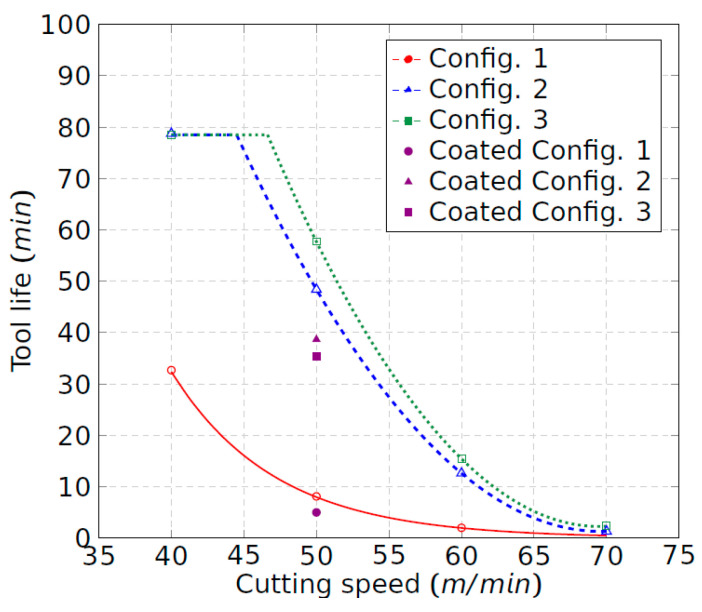
Tool life curves for each configuration.

Taking into consideration the tool wear curves (Figure 3) tool life was obtained by dividing the machining length that promotes a *V_b_* threshold of 0.1 mm by the cutting speed (Figure 5). It is noteworthy that a relevant improvement in tool life was found with the increment of the tool inclination from configuration 1 to configuration 2. Configuration 3 led to an additional moderate enhancement. The influence of the tool inclination is less remarkable as the cutting speed increases. Particularly, the tool life rises by five and six times at 50 m/min for configurations 2 and 3, respectively.

The tool life at 40 m/min, in configurations 2 and 3, is reached at a machining time of 78 min, which is an unexpected result according to the Taylor curve. This means that this value corresponds to the maximum limit of machining time for any speed higher than 40 m/min. The result is consistent with the typical morphology of the edited Taylor curves by tool manufacturers, limiting the field of existence in which the Taylor curves are valid.

For configuration 1, no more than 33 min of machining time could be achieved at the same cutting speed, i.e., 40 m/min. In addition, it can be shown that the tool life for the coated tool at 50 m/min is always lower than the corresponding tool life to the uncoated one for any tool configuration.

### 3.2. Tool Morphology

**Figure 6:**
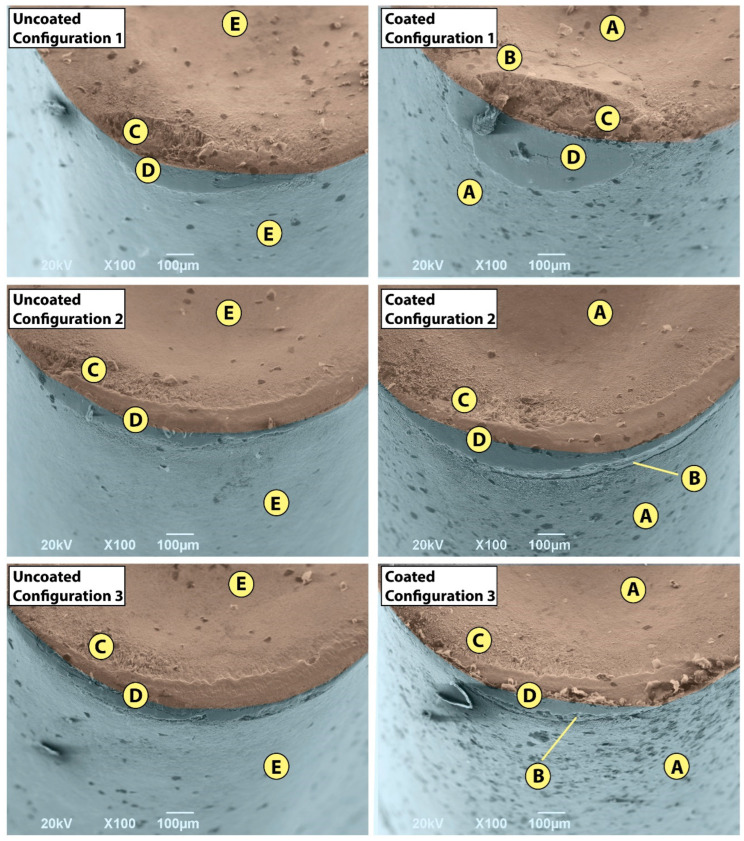
Tool morphology at the end of the tool life for a cutting speed of 50 m/min. A, B, C, D and E point out different regions according to their characteristic chemical composition.

**Figure 7:**
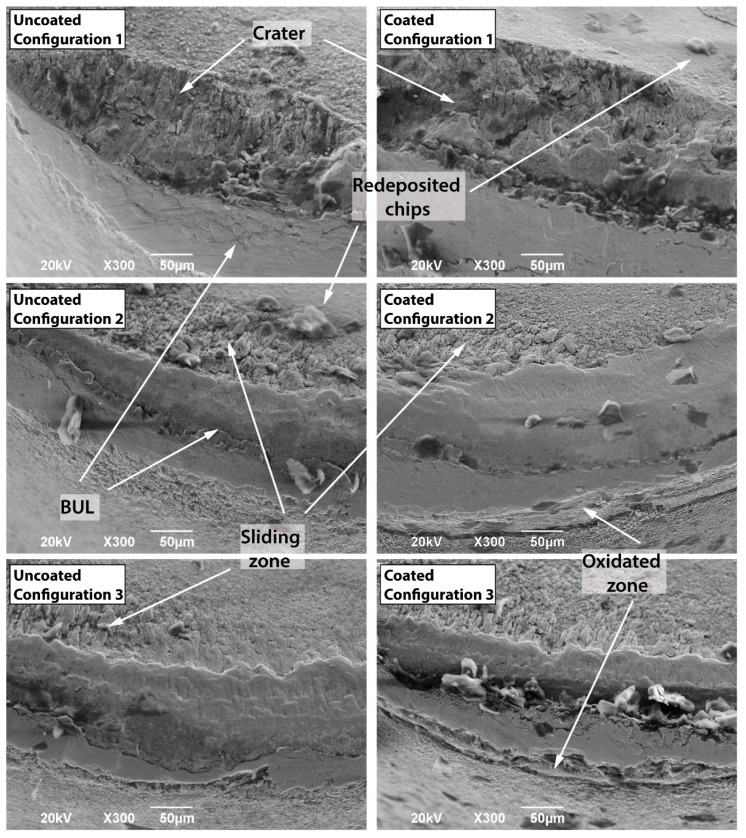
Detail of the wear mechanisms for the experimented coated and uncoated tool after machining at a cutting speed of 50 m/min.

The tool wear morphology at the end of the tool life at 50 m/min is shown in Figure 6, where the two different colors represent the flank and rake faces of the tool. The effect of the tool position configuration on the tool flank and rake faces is significant. Although the wear mechanisms are the same—that is, adhesion of the Ti-aluminide, built-up layer (BUL) formation, abrasion due to high temperatures, as well as chipping of the tool edge—some differences can be observed regarding the tool morphology. According to Figure 6, different zones are distinguished and classified with respect to the chemical composition obtained by EDS analysis (Table 4). Zone A corresponds to the unworn region of the coated insert and the EDS analysis exhibits the typical components that form the coated layers (TiAlN + AlCr_2_O_3_). Region B corresponds to the wear area of the insert in which the coated layer has been removed. This zone is clearly observed in the rake face of the coated tool for configuration 1, where the combination of the temperature and the sliding action of the Ti-aluminide chips removes the coating, reaching the tungsten carbide. In comparison, zone B appears on the flank face for configurations 2 and 3. The presence of oxygen is proof of the high temperatures that remain in the zone due to the low thermal conductivity of the coated layers, leading to oxidation wear [4]. This fact might explain the lower performance observed for the coated tool compared to the uncoated one. In addition, zone E corresponds to the unworn surface for the uncoated tool, which logically, has a different chemical composition (C,W,Co) with respect to zone A of the coated tool. In addition, zone E also differs from B in that it does not have oxygen. Moreover, in zone E, small traces of Ti and Al are found, which can only be justified on the basis of micro-chip adhesions, as indicated in Figure 7. Zones C and D correspond to the chip-tool sliding contact and the BUL area, respectively. Thus, the EDS analysis for zone C shows not only the components that take part in the cutting insert, but also those belonging to Ti-aluminide that are adhered to the tool. This explains that the BUL formed in this region is not continuous, and the chip starts losing contact with the tool. On the other hand, the existence of Ti, Al, Cr and Nb in zone D indicates that the Ti-aluminide deposition on the tool is significant. Eventually, the presence of oxygen indicates that the rubbing zone has been exposed to high temperatures [19].

Regarding the wear mechanisms, configuration 1 (corresponding to the lowest clearance angle), led to wear phenomena on the rake and flank faces, Figure 6, which is clearly relevant. In the flank face, a noticeable adhesive phenomenon of the workpiece material on the tool takes place due to the rubbing effect between the flank wear band and the cylinder, embedding the Ti-aluminide and leading to the BUL formation. The redeposited layer increases with the machining length and with the flank wear, *V_b_*. On the other hand, excessive crater wear occurs in the rake face of the tool, generating a high increment of the cutting forces. The crater wear is proof of the high temperatures and the thermal fatigue that are generated during the cutting process. Zhang et al. [25] stated that the cobalt diffusion on tungsten carbide, produced at high temperatures, dominates the crater-wear mechanism. Therefore, the combination effect of temperature and cutting forces leads to an initial chipping of the tool edge and, as a consequence, a sudden and abrupt failure of the tool, which is consistent with the curves represented in Figure 3a.

With the inclination of the tool in configurations 2 and 3, the tool wear morphology varies significantly. The wear phenomena of the flank and rake faces evolve at the same time, conversely to what happens with configuration 1, where two clearly different zones appear. It is observed that the BUL zone partially covers the rake and flank faces. Combined wear on both faces is slower than when it is produced only on the flank face, which is what makes the tool tip more resistant to mechanical loads. Moreover, the inclination of the tool allows forces and temperatures on the flank face to be reduced. Nevertheless, crater wear was not observed to be produced suddenly, and it might be considered that tool failure mainly appeared due to the progressive evolution of the flank wear band. In addition, the BUL morphology is also different. Therefore, for configuration 1, a simple layer is observed, while several deposited layers are found for configurations 2 and 3 (Figure 7). The non-uniformity of the BUL formation indicates that the layer is deposited and removed sporadically, which is consistent with the results obtained by Anwar et al. [19].

### 3.3. Cutting Forces

**Figure 8:**
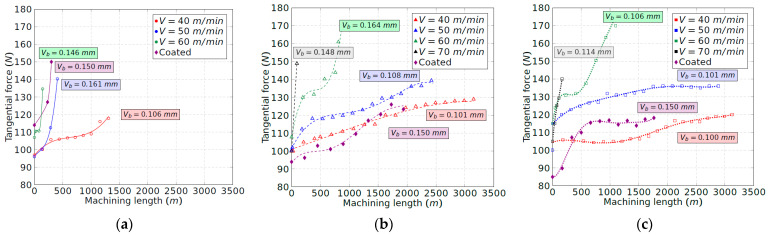
Tangential force evolution vs. machining length: (**a**) tool configuration 1; (**b**) tool configuration 2; and (**c**) tool configuration 3.

**Figure 9:**
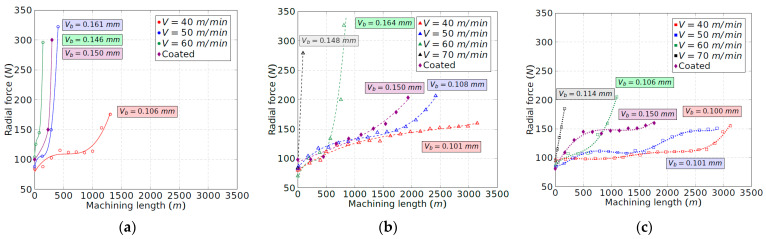
Radial force evolution vs. machining length: (**a**) tool configuration 1; (**b**) tool configuration 2; and (**c**) tool configuration 3.

**Figure 10:**
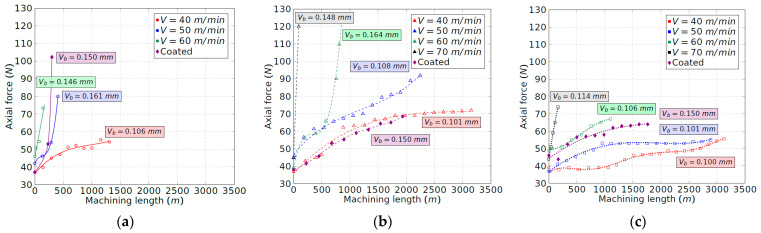
Axial force evolution vs. machining length: (**a**) tool configuration 1; (**b**) tool configuration 2; and (**c**) tool configuration 3.

Figure 8, Figure 9 and Figure 10 show the evolution of the tangential, radial and axial forces for each insert position until the end of the tool life. The speed of 70 m/min is not shown for tool configuration 1 (Figure 8a, Figure 9a and Figure 10a) because the tool broke just at the beginning of the contact with the workpiece. For this research, the machining length equal to 0 m in Figure 8, Figure 9 and Figure 10 aims to describe the forces registered just at the onset of the cutting operation, when the insert is brand new. The initial tangential force for each velocity increased with the increment of the tool inclination, which may be explained according to the reduction in the rake angles, from 9° to 0°, as indicated in Table 2. In comparison, the axial and radial forces are less dependent on the rake angle, with the initial values for each configuration being quite similar.

Comparing the three components of the cutting force, the radial and tangential ones show similar values at the beginning of the machining process for the lowest feed force. A high value of the radial force indicates a poor machinability of the Ti-aluminide as the workpiece tries to repel the tool, which drives a loss of dimensional accuracy in the final part [9]. In addition, the depth of cut selected is small with the aim of avoiding rapid tool wear, which involves a small chip section. This contributes to minimizing the tangential force with respect to the radial one.

Regarding the evolution of the cutting force, the radial component experiences the most remarkable increment with the machining length, reaching values three times higher at the end of the tool life with respect to the beginning of the cutting process. The increment of the radial force is consistent with the evolution of the flank wear band that modifies the tool–workpiece contact area. During the cutting process, the tangential and axial forces also increase by 70% and 140%, respectively. Thus, the tangential force is the least dependent on the tool wear. Nevertheless, in all cases, the evolution of the cutting force components is softer and more gradual with the increment of the tool inclination. According to this, the maximum machining length that can be carried out is higher. On the other hand, the similar shape of the wear and force curves obtained in Figure 3, Figure 8, Figure 9 and Figure 10 indicates a linear relationship between the evolution of the tool flank wear and the cutting forces. These results agree with the work of other authors in similar alloys [26].

Regarding the coated tool, although the initial value of the tangential force is lower in some cases, the increment of the components of the cutting force is faster in comparison to the uncoated tool. Once again, this result is consistent with the curves obtained for the flank wear evolution (Figure 3).

### 3.4. Temperature Analysis

The temperature recorded during the cutting process was within 1030–1070 °C in all cases. This value really corresponds to the temperature in the chip–tool–workpiece area. Nevertheless, these recorded values are near the upper limit for the natural thermocouple to be operative [22]. Thus, it is advisable to analyse the evolution of the temperature with the tool wear without considering the feed rate. Under these conditions, temperature is only generated by the friction phenomenon in the tool–workpiece contact, which is defined by the flank wear band of the tool, since no chip is being removed.

The temperature in the tool–workpiece contact zone depends on the heat power, *H*, whose result can be interpreted as the product of the friction force in the flank wear zone, *F_f_*, and the cutting speed, *V*. In previous studies, it has been demonstrated that the friction coefficient is very high on the machining of titanium aluminides [17]. Taking into account that the tool–workpiece contact area, defined by the flank wear, *V_b_*, is lower than 0.1 mm, the heat intensity is very high in the mentioned area. A high temperature is definitely expected at the tool tip, especially considering the poor thermal diffusivity of Ti-aluminide alloys.

**Figure 11:**
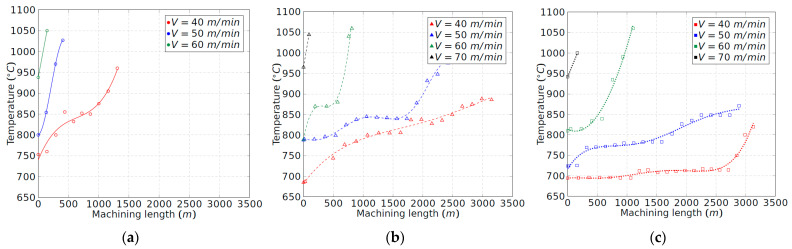
Tool–workpiece contact temperature due to the friction phenomenon in the flank wear zone: (**a**) tool configuration 1; (**b**) tool configuration 2; and (**c**) tool configuration 3.

The experimental results for the temperature under the explained conditions are collected in Figure 11. As expected, the temperature always increases with the cutting speed. The results depicted in Figure 11 agree with a rapid flank wear phenomenon produced at higher temperatures than 900 °C. Wear is produced by combining thermal and friction phenomena at the tool tip, promoting the embrittlement of the binder phase of the cutting tool. The oxidation of the cobalt phase has effectively been reported in literature [27]. As observed, in Figure 11b,c, the temperature for 70 m/min is always greater than 900 °C, leading to a catastrophic failure for each tool configuration.

Eventually, the inclination of the cutting insert, which means a progressive increment of the clearance angle for configurations 2 and 3, helps to reduce the tool–workpiece contact temperature as the friction force reduces in size in the flank zone. It can be observed that not only the initial temperature has been reduced, but also the slopes of the temperature curves. Thus, in configuration 1, the limit temperature of 900 °C is reached for all cutting speeds; meanwhile in configurations 2 and 3, that limit is not reached for the lowest experimented speeds. Last, but not least, it must be mentioned that the procedure for measuring the temperature involved was not carried out with chip removal, which allows us to evaluate the improvement of the machining process with the tool configuration.

### 3.5. Surface Integrity

**Figure 12:**
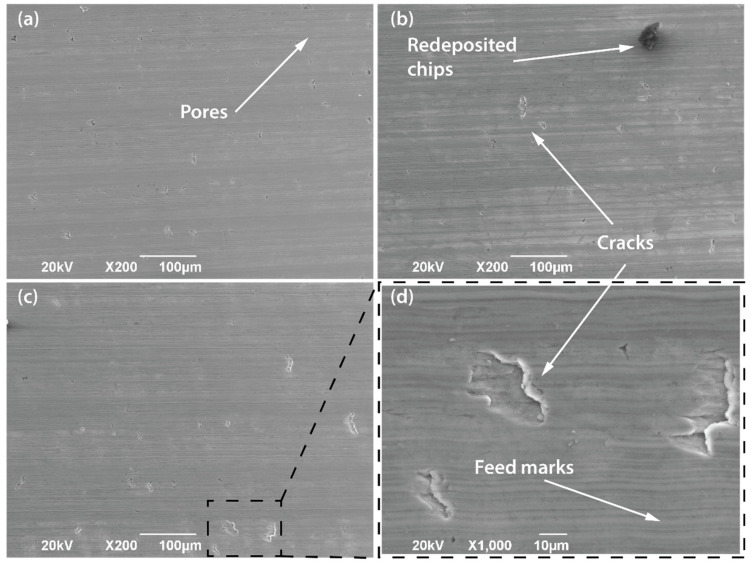
Morphology of the workpieces machined by using the uncoated tool at a cutting speed of 50 m/min: (**a**) configuration 1; (**b**) configuration 2; (**c**) configuration 3; and (**d**) detail of the cracks induced by machining for configuration 3.

Figure 12 shows the surface morphology of the machined workpieces with the uncoated tool for the different tool configurations at a cutting speed of 50 m/min. Representative feed marks are visible for all conditions. Although the machined surfaces seem to be relatively undamaged, some visible defects are observed. Thus, some surface microcracking appears due to the high forces during the turning process along with the brittle nature of the titanium aluminide. Moreover, as was mentioned, the effect of the radial force is to reject the tool from the workpiece surface, promoting vibration effects that contribute to causing the phenomenon. In addition, a few small pores due to the manufacturing process can be found.

Comparing the three tool configurations considered herein, the density of surface cracks decreases with the inclination of the cutting tool. However, for configurations 2 and 3 the size of the surface cracks is bigger than in configuration 1.

**Figure 13:**
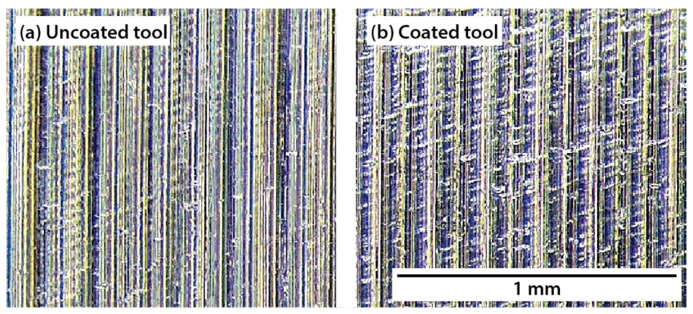
Machined surfaces with the (**a**) uncoated and (**b**) coated tool for configuration 3 at 50 m/min.

Figure 13 shows the surfaces machined with an uncoated and a coated tool. The number of surface defects is more significant for the uncoated tool. Therefore, thick feed marks and a remarkable grooved morphology can be observed. This poor surface may be produced as an effect of the excessive heat generated during the cutting process, which remains at the tool–workpiece contact interface because of the poor thermal conductivity of the coatings layers [24]. Under this condition, diffusion phenomena promote surface damage of the workpiece. In addition, these results are consistent with those obtained by the authors [19].

**Figure 14:**
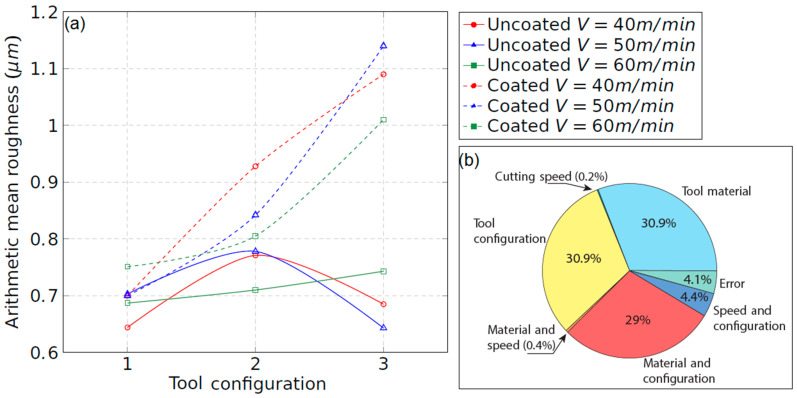
(**a**) Arithmetic mean roughness of the machined surface vs. tool configuration; (**b**) contribution of each parameter on surface roughness obtained from an ANOVA analysis.

Figure 14a shows the arithmetic mean roughness, Ra, of the machined surfaces at the beginning of the turning process, that is, under the new tool condition. With the objective of developing an analysis of variance (ANOVA) of all cutting factors affecting surface roughness, specific short tests were carried out at each cutting speed for both tools. The three variables considered were the tool configuration, described by the back clearance angle (αy) the cutting speed (V) and the tool material (M). Three levels were taken into account for the two first variables, and two for the tool material, assigning zero for the uncoated tool and one for the coated one, respectively (Table 5). ANOVA results, developed with a confidence level defined by R2 = 0.958, can be observed in Table 6 and Figure 14b. The Ra values for the coated tool are greater than for the uncoated one, especially with the increment of the tool inclination. This is indicative of the poor surface quality of parts machined with this tool with respect to an uncoated one.

For the uncoated tool, no correlation of Ra with the tool configuration was found. Conversely, the coated tool shows worse behaviour that is clearly influenced by the tool configuration. Particularly, configuration 3 leads to the worst surface finish. On the other hand, the cutting speed has a low influence on the surface roughness, as shown in the ANOVA analysis in Figure 14b, which is typical for brittle materials [18].

To sum up, for both tools, the configuration, the tool material and their combination effect were found to be the most relevant parameters that affect the surface roughness change, according to the ANOVA analysis.

### 3.6. Subsurface Microhardness and Microstructure

**Figure 15:**
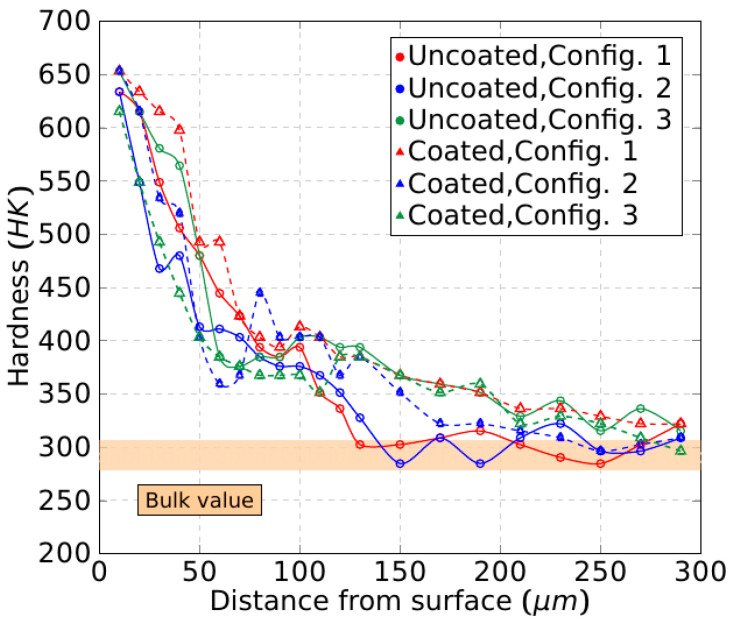
Knoop microhardness profile of the machined workpiece.

Figure 15 shows the subsurface microhardness profile of the machined workpieces, starting with a new tool for each test at a cutting speed of 50 m/min. An important hardening effect is observed near the machined surface, twice as much as the bulk material hardness. This is a result of the mechanical work during the cutting operation in spite of the thermal effects induced by the high cutting temperatures. Therefore, it indicates the sensitivity of the Ti-aluminides to strain hardening, which reduces surface ductility [28].

The nearest hardness print from the surface that can be carried out is located at 10 µm and, at this point, the hardness is twice that of the bulk one. The hardness decreases rapidly through the inner material until 80 µm deep. Then, it is reduced more progressively up to the typical bulk value, which is found at 300 µm deep. Similar profiles are observed for the three tool configurations studied, which means that this feature does not influence the subsurface. On the other hand, the coating of the tool is not relevant in this context. Another aspect to be pointed out is the high absolute hardness values obtained close to the surface of the workpiece, that are probably near the plastic limit capacity. The surface microcracking phenomenon indicated above enhances this hypothesis.

**Figure 16:**
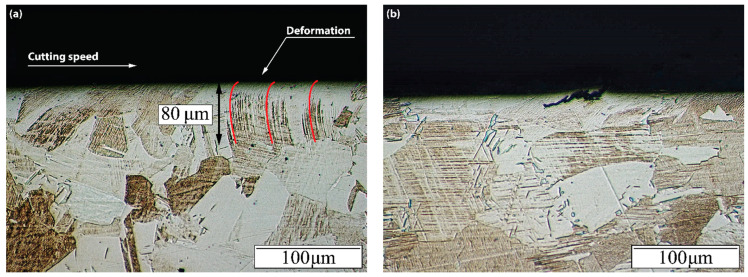
(**a**) Typical microstructure alterations and deformed layer induced by machining (configuration 1 and 50 m/min cutting speed); (**b**) detail of surface cracks (configuration 2 for a cutting speed of 50 m/min).

In addition, a microstructural change can be observed, which is depicted in Figure 16a. The deformation of γ + α_2_ lamellas is produced along the cutting speed direction, which indicates strain existence at high temperatures [29]. The deformed layer depth is roughly 60–80 µm, which is coherent with the microhardness profile obtained. Once again, no influence of the coating or the tool configuration was detected. In Figure 16b, a detail of the surface microcracks can be seen, around 15 µm deep. This reinforces the statement of plastic depletion of the material.

## 4. Conclusions

In this study, the turning process of Ti48Al2Cr2Nb aluminide under sustainable dry conditions was comprehensively evaluated. It was demonstrated that a simple technical solution, based on the change of the tool configuration, clearly increases the efficiency of the operation, ensuring the sustainability of the operation. Thus, the effect of the tool inclination in terms of tool wear, tool life, cutting forces, cutting temperature, surface integrity and subsurface microhardness and microstructural modification was analysed. In addition, the effectiveness of an uncoated carbide tool and a PVD TiAlN + AlCr_2_O_3_ coated one was assessed. More detailed results are summarised as follows:

1. Cutting speed plays a critical role in controlling the performance of the cutting process. As expected, the increment of the cutting speed generates an excessive temperature that accelerates abrasion. For the lowest tool inclination, machining with a cutting speed of 40 m/min is stable until a 1000 m of machining length. The increase in cutting speed for 50 m/min, 60 m/min or 70 m/min leads to accelerated crater wear with a dramatic failure of the tool.
2. The EDS analysis of the tool wear morphology shows the strong tendency of the γ-TiAl to adhere to the tool, especially to the flank face. With the lowest inclination of the tool, the BUL formation totally covers the flank face, while the increment of the tool inclination promotes combined wear in the flank and rake faces, which reduces the tool wear rate.
3. The inclination of the cutting insert, which increases the clearance angle between the tool flank face and the workpiece, positively affects the tool wear evolution, although the tangential force is higher due to the reduction in the rake angle. This is explained according to the reduction in the cutting temperature and friction forces in the flank face of the tool. In addition, the inclination of the tool makes the tool tip geometrically more resistant. Therefore, flank wear is under control, and crater wear is avoided. As a result, higher tool life is obtained and machining operations at 50 m/min and 60 m/min become possible. Particularly, the tool life rises by five and six times at 50 m/min for configurations 2 and 3, respectively. Machining at 60 m/min implies a 50% increment of the cutting speed without the need to use lubricants.
4. The uncoated tool performs better than the coated one in terms of tool wear and tool life for all cutting speeds. It is explained according to the high temperature reached in the tool flank area due to the lower thermal diffusivity of the coating layer. The oxidation wear found in the coated tool supports this result.
5. Although the surface of the machined parts is mostly undamaged, proof of surface microcracks is found for all tool configurations. The density of cracks decreases with the inclination of the tool, but their size is larger. Comparing both inserts, the surface integrity of the machined parts with the coated tool shows a worse appearance with a higher number of defects and thick feed marks.
6. The surface roughness obtained in the machined parts with the uncoated tool, which are within 0.65–0.75 µm, is lower than that of a coated one. The coating of the tool and its inclination are found to be the most remarkable factors in the variation in surface roughness. Furthermore, the cutting speed does not have an influence on surface roughness according to the ANOVA analysis.

## Figures and Tables

**Table 1:** Ti48Al2Cr2Nb chemical composition (wt%) from supplier.

Ti	Al	Nb	Cr	C	H	N	O
59.10	33.45	4.72	2.51	0.005	0.001	0.007	0.045

**Table 2:** Cutting tool angles according to the machine reference system.

Designation	Back Rake Angle, γ_y_	Side Rake Angle, γ_x_	Back Clearance Angle, α_y_	Side Clearance Angle, α_x_
Config. 1 (original shim)	9°	9°	6.3°	6.3°
Config. 2	3.7°	6°	11.6°	9.4°
Config. 3	0°	3°	15°	12.3°

**Table 3:** Cutting parameters for the experimental tests.

Cutting Parameters	Uncoated TNMG 160408-SM H13A	PVD Coated TNMG 160408-SM 1115
Feed rate (mm/rev)	0.1	0.1
Depth of cut (mm)	0.25	0.25
Cutting speed (m/min)	40, 50, 60, 70	50

**Table 4:** Chemical composition of each zone determined by EDS analysis.

Zone.	Tool	Element Weight (%)								
		C	Co	W	Ti	Al	Cr	Nb	N	O
A	Coated, 1	-	-	-	37.56	22.63	9.48	-	12.63	17.71
B	Coated, 1	26.76	6.42	50.55	1.17	-	0.65	-	-	14.45
C	Uncoated, 3	-	2.18	36.56	16.05	9.33	-	-	-	35.88
D	Uncoated, 2	-	-	-	54.58	32.14	2.18	4.80	-	6.29
E	Uncoated, 1	35.23	3.93	56.43	2.65	1.76	-	-	-	-

**Table 5:** Values of the factors used in the ANOVA analysis.

Factor	Levels	Values
Cutting speed, V (m/min)	3	40, 50, 60
Tool configuration, α_y_ (°)	3	6.3, 11.6, 15
Tool material, M	2	0, 1

**Table 6:** ANOVA analysis results for R_a_.

Factor	DF	Adj. SS	Adj. MS	F	P	Contribution (%)
M	1	0.1261	0.1261	30.01	0.005	30.9
V	2	0.0009	0.0004	0.11	0.895	0.2
α_y_	2	0.1262	0.0631	15.01	0.014	30.9
M · V	2	0.0015	0.0007	0.19	0.838	0.3
M · α_y_	2	0.1183	0.0591	14.07	0.015	29.0
V · α_y_	4	0.0179	0.0044	1.07	0.476	4.4
Error	4	0.0168	0.0042	-	-	4.1
Total	17	0.4079	-	-	-	100

## Data Availability

Not applicable.
